# A Novel Chemically Modified Curcumin Reduces Severity of Experimental Periodontal Disease in Rats: Initial Observations

**DOI:** 10.1155/2014/959471

**Published:** 2014-06-29

**Authors:** Muna S. Elburki, Carlos Rossa, Morgana R. Guimaraes, Mark Goodenough, Hsi-Ming Lee, Fabiana A. Curylofo, Yu Zhang, Francis Johnson, Lorne M. Golub

**Affiliations:** ^1^Department of Oral Biology and Pathology, School of Dental Medicine, Stony Brook University, Stony Brook, NY 11794, USA; ^2^Department of Diagnosis and Surgery, School of Dentistry at Araraquara, UNESP, 14801-903 Araraquara, SP, Brazil; ^3^Department of Chemistry, Stony Brook University, Stony Brook, NY 11794-3400, USA

## Abstract

Tetracycline-based matrix metalloproteinase- (MMP-) inhibitors are currently approved for two inflammatory diseases, periodontitis and rosacea. The current study addresses the therapeutic potential of a novel pleiotropic MMP-inhibitor not based on an antibiotic. To induce experimental periodontitis, endotoxin (LPS) was repeatedly injected into the gingiva of rats on one side of the maxilla; the contralateral (control) side received saline injections. Two groups of rats were treated by daily oral intubation with a chemically modified curcumin, CMC 2.24, for two weeks; the control groups received vehicle alone. After sacrifice, gingiva, blood, and maxilla were collected, the jaws were defleshed, and periodontal (alveolar) bone loss was quantified morphometrically and by *μ*-CT scan. The gingivae were pooled per experimental group, extracted, and analyzed for MMPs (gelatin zymography; western blot) and for cytokines (e.g., IL-1*β*; ELISA); serum and plasma samples were analyzed for cytokines and MMP-8. The LPS-induced pathologically excessive bone loss was reduced to normal levels based on either morphometric (*P* = 0.003) or *μ*-CT (*P* = 0.008) analysis. A similar response was seen for MMPs and cytokines in the gingiva and blood. This initial study, on a novel triketonic zinc-binding CMC, indicates potential efficacy on inflammatory mediators and alveolar bone loss in experimental periodontitis and warrants future therapeutic and pharmacokinetic investigations.

## 1. Introduction

Over the past several decades, numerous studies have described pharmacologic strategies to utilize matrix metalloproteinase-inhibitors (MMP-Is) to prevent connective tissue breakdown associated with various inflammatory and other diseases, for example, periodontitis, arthritis, osteoporosis, cardiovascular disease, and cancer [1–4]. Recently, these have also included less obvious strategies such as (but not limited to) blocking MMP-mediated cleavage of insulin receptors in type-2 diabetics to improve insulin sensitivity [5] and to reduce HbA1c levels [6]. However, to date, the only orally (systemically) administered MMP-Is approved by the US-FDA and other national regulatory agencies (Europe and Canada) are those based on the surprising nonantimicrobial properties of the tetracycline antibiotics [4, 7–9]. In this regard, studies on experimental animals and on human subjects have demonstrated the efficacy of nonantimicrobial tetracycline formulations, as pleiotropic MMP-Is, in periodontal and other diseases [4, 7, 9, 10]. In addition to demonstrating that these medications, which include two formulations of subantimicrobial-dose doxycycline (both FDA-approved), can inhibit collagenolysis, connective tissue destruction, and bone resorption in the diseased periodontal tissues, other therapeutic mechanisms have also been identified. These include suppressed expression of inflammatory mediators such as the cytokines (e.g., IL-1*β*, TNF-*α*, and IL-6), prostaglandins, reactive oxygen species (e.g., HOCl), and nitric oxide, the latter reflecting the inhibition of inducible nitric oxide synthase [11, 12].

Given this background, a search has been underway for new drug molecules which exhibit a similar active site for MMP-inhibition as the tetracyclines “but with a different phenolic superstructure” [11]. With this strategy in mind, the therapeutic potential of the tetracycline diketonic, metal-ion binding site [8, 9] has been expanded by the recent development of a new series of compounds with a similar zinc-binding moiety, which are bicyclic rather than tetracyclic, that is, the chemically modified curcumins or CMCs. The structures of these compounds, their potency and mechanisms of action as MMP-Is, and their zinc-binding (and other) characteristics have been described recently, and a “lead” compound has been identified [11, 13, 14]. This compound, CMC 2.24, is a phenylamino carbonyl curcumin, is triketonic (which enhances its zinc-binding characteristics) in contrast to the diketonic active site on both the tetracyclines and on traditional/natural curcumin compounds, and has shown evidence of efficacy in vitro, in cell and organ culture, and in animal models of chronic inflammatory and other diseases [13–15]. As additional background, recent studies have shown that natural/unmodified curcumin administered to rats with experimentally induced periodontal disease was effective in reducing inflammatory mediators and MMPs in the gingiva and periodontal ligament but was ineffective in reducing the excessive resorption and loss of alveolar bone [16]. Accordingly, the current report describes the first of a series of studies which examined the efficacy of CMC 2.24 as a pleiotropic MMP-I in several rat models of periodontitis with a particular focus on its ability to inhibit pathologic alveolar bone loss. Moreover, because of the long-standing interest in the link between the oral disease, periodontitis, and systemic inflammation (the latter associated with increased risk for various diseases, notably cardiovascular disease and more severe diabetes [4, 17]), the effects of treatment with this novel compound on biomarkers in the circulation were also examined.

## 2. Materials and Methods

### 2.1. Experimental Periodontal Disease Model

**Figure 1:**
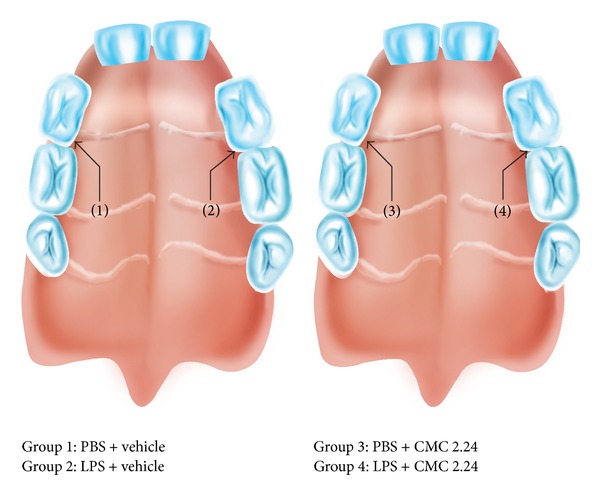
Diagrammatic representation of the four experimental groups using “split-mouth” protocol.

Eleven male Holtzman rats (*Rattus norvegicus albinus*) weighing 150–250 g were maintained under pathogen-free conditions with controlled temperature (21 ± 1°C) and humidity (65–70%) and a 12 h light-dark cycle. Food and water were provided* ad libitum* throughout the experiment. General anesthesia was induced by inhalation of an isoflurane/oxygen mixture. 30 *μ*g of lipopolysaccharide (LPS) from* Escherichia coli* (strain 055:B5; Sigma Chem Co., St. Louis, MO, USA) diluted in phosphate buffered saline (PBS) was injected into the palatal gingiva (3 *μ*L volume per injection) using a Hamilton microsyringe (Agilent, Santa Clara, CA, USA) as described by us previously [18]. These LPS injections were made into the palatal tissue between the upper 1st and 2nd molars, on the left side of the animal, three times a week for 14 days (a total of 6 injections and 180 *μ*g of LPS in each site). The opposite side received injections of the same volume of PBS vehicle and served as the control site (“split-mouth” protocol; see Figure 1). At the end of the experimental period, the animals were sacrificed by CO_2_ inhalation and samples were collected as described below. Also at the time of sacrifice, blood samples were collected and the serum and plasma were separated by standard procedure and analyzed for MMPs and cytokines as described below. The study protocol was previously approved by the Institution's Committees (Araraquara-UNESP, SP, Brazil, and Stony Brook University, NY, USA) for Experimental Animal Use.

### 2.2. Experimental Groups

The effects of CMC 2.24 (a phenylamino carbonyl curcumin) were assessed in a “prophylactic” model (the efficacy of this compound in a “therapeutic” model will be assessed in future studies) in which the induction of periodontal disease by LPS injections was carried out during the same period of time (14 days) as the daily oral administration of CMC 2.24 (30 mg/kg) or the vehicle-control. The test compound and the vehicle-control (a 1 mL suspension of 2% carboxymethyl cellulose) were both administered once per day over the 14-day protocol by oral intubation. The rats and their periodontal tissues were randomly distributed into the following experimental groups as illustrated in Figure 1.

Group 1—gingiva injected with PBS in rats systemically administered vehicle alone (*n* = 5); group 2—gingiva injected with* E. coli* LPS in the vehicle-treated rats (*n* = 5) (note: with this “split-mouth” design, group 1 and group 2 tissues involve the same 5 rats); group 3—gingiva injected with PBS in rats systemically administered the test medication (CMC 2.24; *n* = 6); and group 4—gingiva injected with* E. coli* LPS in rats systemically administered CMC 2.24 (*n* = 6) (as above, groups 3 and 4 involve the same 6 rats). However, for the *μ*-CT analysis, additional rats were added to each experimental group resulting in *n* = 10 rats per group.

### 2.3. Gingival Tissue Extract and Its Partial Purification

The gingival tissues from the hemimaxilla of each rat were excised and pooled per experimental group (5-6 rats per group) as described by us previously [19, 20]. The pooling of gingival tissues for each group was necessary because individual rats do not yield sufficient gingiva for enzyme analyses. The gingival tissues were extracted and the MMPs were partially purified as described by us previously [19, 20]. In brief, the samples were homogenized (all procedures at 4°C) with a glass grinder (Kontes, Glass Co., Vineland, NJ) attached to a T-Line Lab stirrer (Model 106 Taboys Engineering Corp., NJ) in 50 mM Tris-HCl buffer (pH 7.6) containing 5 M urea, 0.2 M NaCl, and 5 mM CaCl_2_ and then extracted overnight and centrifuged at 15,000 rpm for 1 h. The supernatants were collected and dialyzed exhaustively against 50 mM Tris buffer (pH 7.8) containing 0.2 M NaCl and 5 mM CaCl_2_. Ammonium sulfate was added to the dialysate to produce 60% saturation, allowed to stand overnight, and the precipitate containing the MMPs was collected by centrifugation at 15,000 rpm for 90 min. The pellets were then dissolved in the Tris buffer (pH 7.8) containing NaCl, CaCl_2_, and 0.05% Brij and exhaustively dialyzed against the same buffer. Protein content of the extracts was determined by Bio-Rad Protein Assay.

### 2.4. Zymographic Assay of MMP-2 (Gelatinase A) and MMP-9 (Gelatinase B)

**Figure 2:**
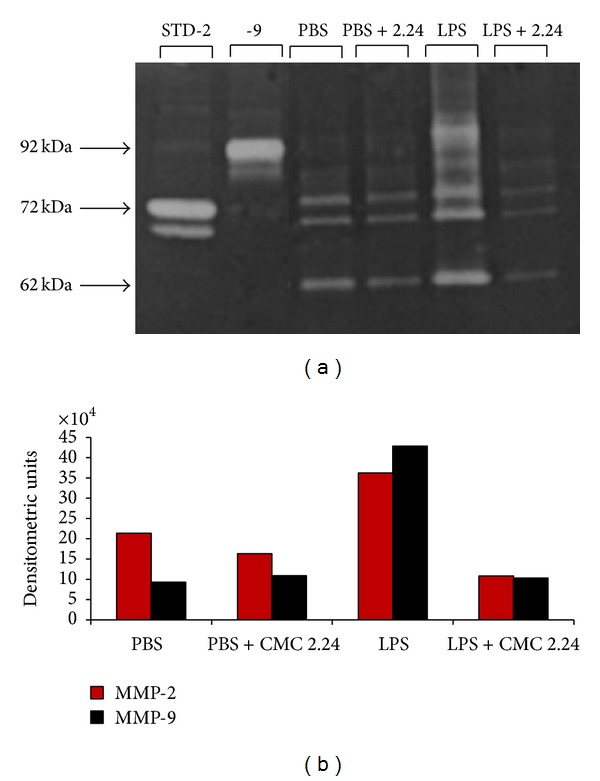
(a) Gelatin zymography of partially purified extract of gingiva from each experimental group showing the effect of orally administered CMC 2.24 on gingival MMPs (-2, -9). In groups 1 and 3, all rats received PBS injections into the gingiva plus oral administration of either vehicle alone (group 1) or CMC 2.24 (group 3). In groups 2 and 4, all rats received LPS injections into the gingiva plus oral administration of either vehicle alone (group 2) or CMC 2.24 (group 4). (b) Densitometric analysis of gingival MMPs (-2, -9).

The relative levels of the higher molecular weight proforms and the lower molecular weight activated forms of MMP-2 and MMP-9, in the pooled gingival extracts from each of the four experimental groups (Figure 2), were determined by zymography (the gelatin zymography system was purchased from Invitrogen Corp., Carlsbad, CA). In brief, all samples were run under nonreducing denaturing conditions on the gelatin zymography system containing polyacrylamide copolymerized with gelatin at a final concentration of 1 mg/mL. After electrophoresis, the gels were washed in 2.5% Triton X-100 and incubated at 37°C overnight in the assay buffer (40 mM Tris, 200 mM NaCl, and 10 mM CaCl_2_; pH 7.5). After incubation, the gels were stained with SimplyBlue SafeStain (Invitrogen Corp., Carlsbad, CA). Clear zones of lysis against a blue background indicate gelatinolytic activity, as described by us previously [11, 21, 22]. Densitometric analysis of the gelatinolytic bands was carried out using the Scientific Imaging system (KODAK ID 3.5, Rochester, NY).

### 2.5. Alveolar Bone Loss Measurements

Since this is a major outcome in the experimental periodontal disease model and since reducing alveolar bone loss is a key therapeutic goal in treating human inflammatory periodontal disease, two methods were used to assess the effects of CMC 2.24 on this inflammatory-driven bone loss model.

#### 2.5.1. Morphometric Analysis of Alveolar Bone Loss

As described previously [23], the soft tissues were carefully dissected to maintain the integrity of the maxillary bone specimens. These were then completely defleshed by immersion in 8% sodium hypochlorite for 4 h followed by gentle mechanical scavenging of the remaining soft tissue. After washing in running water, the specimens were immediately dried with compressed air. To distinguish the cementum-enamel junction (CEJ), 1% aqueous methylene blue solution (Sigma-Aldrich, Saint Louis, MO, USA) was applied to the specimens for 1 min and then washed in running water. The specimens were fixed on 3 mm thick red dental wax with their palatal surface facing up. Standardized orientation was achieved by positioning the specimens with the palatal cusp tip of the first and second molars superimposed on the corresponding buccal cusp tips (i.e., occlusal plane perpendicular to the ground). To validate measurement conversions, a millimeter ruler was positioned on the wax and photographed with all specimens. The specimens were positioned under a stereomicroscope (Leica MZ6, Buffalo Grove, IL, USA) and digital images were obtained at 25x magnification using a 6.1-megapixel color digital camera coupled to the microscope.

A single examiner, who was not aware of the experimental group allocation of the specimens, carried out all morphometric measurements of alveolar bone loss by delineating the area of exposed root surface of the first and second molars using an image analysis software (Leica Application Suite, v3.8.0, Leica Microsystems, Buffalo Grove, IL, USA) and the results were converted to mm^2^ using measurement of the reference millimeter grid. The area of exposed root surface in each specimen was averaged according to the experimental groups. Intraexaminer calibration was performed by evaluating repeated measurements of 10 nonstudy images presenting alveolar bone loss similar to the present study. The intraclass correlation showed a 96.8% reproducibility.

#### 2.5.2. Microcomputerized Tomography (*μ*-CT)

Upon sacrifice, the hemimaxillae of the rats were dissected including teeth and surrounding soft tissues, fixed for 18–24 h in 10% neutral buffered formalin at 4°C, washed in distilled water, and transferred to 70% ethanol. This procedure allowed us to use these same specimens for the histological assessments used in subsequent studies (Guimaraes et al., in preparation). These samples were scanned on a microcomputer tomograph (Skyscan 1176, SkyScan, Aartselaar, Belgium) using 18 *μ*m slices. The digital radiographic images of each sample were reconstructed into a three-dimensional model (NRecon Software, SkyScan, Aartselaar, Belgium) consisting of a matrix of 18 × 18 × 18 *μ*m and a standardized gray scale value to visualize only mineralized tissues. Using the software package Dataviewer*∖*CTan*∖*CTvol (Skyscan, Aartselaar, Belgium), the reconstructed tridimensional matrix of each sample was initially reoriented in a standardized manner on three planes: sagittal, coronal, and transversal. Subsequently, a cubic region of interest (ROI) of 9.72 mm^3^ was defined using standardized dimensions and anatomical landmarks: cementum-enamel junction of the first molar as the coronal limit extending vertically 1.5 mm apically, an anteroposterior dimension of 3 mm from the distal aspect of the mesial root of the first molar, and the transversal (buccolingual thickness) dimension of 2.16 mm (120 slices of 18 *μ*m each). This ROI included the first molar, half of the second molar, and also approximately 1 mm from the most palatal aspect of the first molar crown (including the palatal bone adjacent to the first and second molar teeth which was the site of LPS injections). We determined the relative volume of this ROI occupied by mineralized tissue in each sample. The data was averaged for each experimental group and compared by nonpaired *t*-tests using Welch's correction for unequal variances. Significance level was set to 95%.

### 2.6. Immunoblotting for Measurement of MMP-8 in Plasma and Gingival Extracts

MMP-8 levels in plasma and gingival extracts, the latter prepared as described above, were determined by western blot analysis. In brief, samples were reduced, boiled, subjected to SDS/PAGE, and transferred to polyvinylidene difluoride (PVDF) membrane (Amersham Pharmacia Biotech Inc., Piscataway, NJ). Blots were blocked with 5% nonfat dry milk for 2 h at room temperature. The membranes where then incubated with polyclonal antibodies specific for MMP-8 (Abcam PLC, Cambridge, MA) overnight at 4°C. Blots were washed and incubated with secondary antibodies purchased from Thermo Scientific for 2 h at room temperature. Detection of the bands was carried out on radiographic film by using SuperSignal West Dura Extended Duration Chemiluminescent substrate (Thermo Fisher Scientific Inc., Waltham, MA). The band densities were quantified by scanning on a laser densitometer [24]. To assess the levels of inactive (proform) and smaller molecular weight active forms of the MMP-8 (collagenase-2), bands corresponding to both molecular weight forms were quantitated, and the data is expressed as densitometric units and as the ratios of inactive/active forms. Recombinant rat MMP-8 (source: mouse myeloma cell line, NSO derived) from R&D Systems (Minneapolis, MN) was used as a standard for western blot analysis of the rat plasma samples. This MMP-8 standard was incubated for 4 hours at room temperature, in the presence or absence of 1 mM amino phenyl mercuric acetate (APMA), a known activator of higher molecular weight pro-MMPs into the lower molecular weight activated forms [20].

### 2.7. ELISA for Measurement of MMP-13 in Plasma and Gingival Extracts

The level of MMP-13 was measured in the gingival tissue extracts and plasma of each rat by enzyme-linked immunosorbent assay (ELISA). This assay was performed according to the manufacturer's instructions (TSZ Scientific LLC, Framingham, MA). Blood samples from animals in each experimental group were assayed in duplicate.

### 2.8. Measurement of Gingival Tissue and Serum Levels of Bone Resorptive Cytokines

The levels of 3 bone resorptive cytokines (IL-1*β*, IL-6, and TNF-*α*) were measured in serum and gingival tissue extracts by enzyme-linked immunosorbent assays (ELISAs). These assays were performed according to the manufacturer's instructions (R&D systems, Minneapolis, MN), and the results were normalized to the total concentration of protein in the samples. Blood samples from animals in each experimental group were assayed in duplicate.

## 3. Results

### 3.1. Local/Oral Measurements: Gingiva and Alveolar Bone

The levels of both MMP-2 (72 kDa progelatinase) and MMP-9 (92 kDa progelatinase) were assessed by gelatin zymography in pooled gingival tissue from half-jaws of rats from each experimental group (Figure 2). LPS-induced periodontal disease dramatically increased MMP-2 and MMP-9 levels in the pooled gingival tissue, while lower levels of the pro- (higher molecular weight) and activated (lower molecular weight) forms of these gelatinases were seen in the gingival tissue from all of the other experimental groups. Treatment of the rats with systemically administered CMC 2.24 appeared to “normalize” the pathologically excessive levels of the various molecular weight forms of these gelatinolytic MMPs in the LPS-injected gingiva assessed either visually (Figure 2(a)) or by densitometric analysis of the zymograms (Figure 2(b)). Some reduction of these MMP proteinases by CMC 2.24 administration was also seen in the gingiva from the rats without LPS injections (Figures 2(a) and 2(b)).

**Figure 3:**
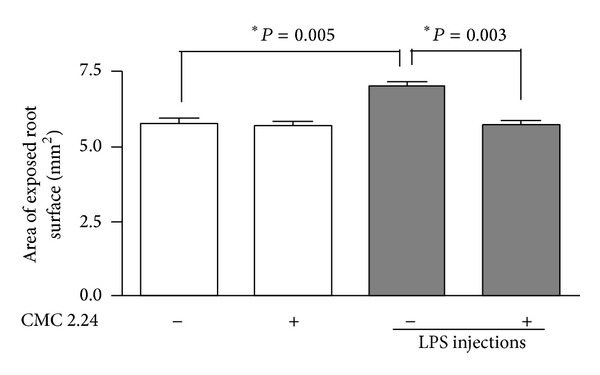
Direct measurements on defleshed hemimaxillae demonstrate that CMC 2.24 significantly inhibits alveolar bone resorption in the in vivo model of LPS-induced periodontal disease. The bar graph presents the results of the percent of exposed root surface, which is directly proportional to the extent of bone loss, according to the experimental group. LPS caused bone loss as indicated by the significant increase of the area of exposed root surface, whereas simultaneous systemic administration of CMC 2.24 significantly reduced this area, indicating an attenuation of inflammatory-driven bone resorption. Differences between experimental conditions are indicated by the brackets and ∗ (unpaired *t*-test for independent samples with Welch's correction for unequal variances).

In a pattern reminiscent of the zymograms described above and based on morphometric analysis of alveolar bone height loss which measured the area of exposed root relative to the cementoenamel junction as a fixed anatomical landmark, LPS injections into the gingiva significantly (*P* = 0.005) increased alveolar bone loss (Figure 3). Moreover, when the LPS-injected rats were treated by oral administration of CMC 2.24, alveolar bone loss was significantly reduced (*P* = 0.003) back to the normal level seen in the rats not exposed to gingival LPS injections. Note that CMC 2.24 treatment did not affect alveolar bone loss in the control rats receiving injections of PBS vehicle rather than LPS (Figure 3).

**Figure 4:**
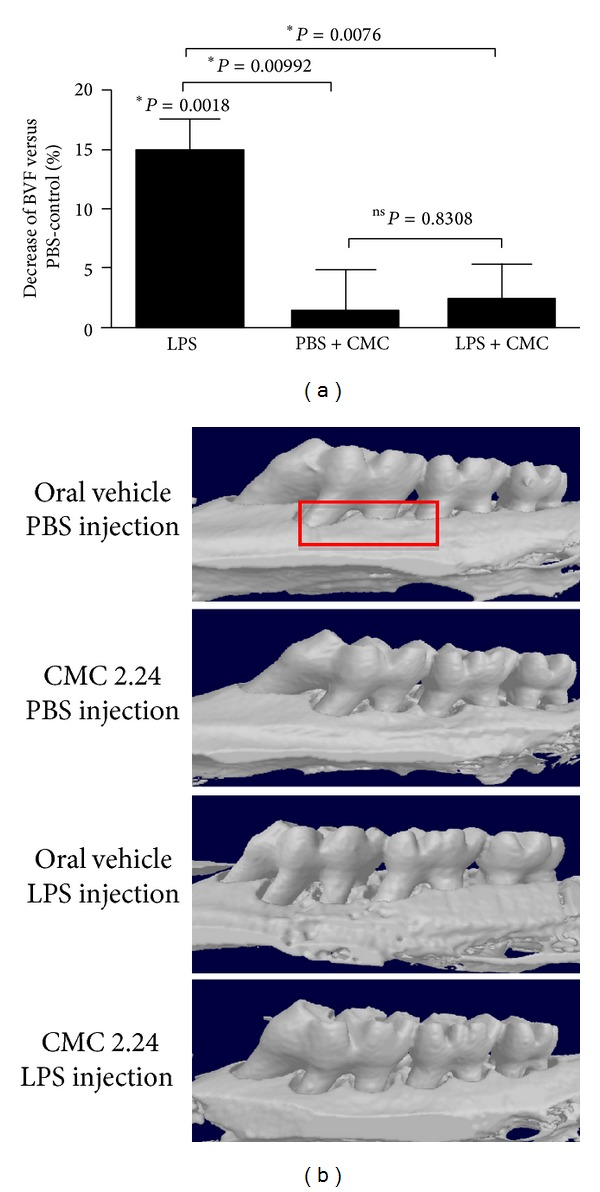
*μ*-CT data confirming that CMC 2.24 significantly inhibits alveolar bone resorption in the in vivo model of LPS-induced periodontal disease. Rats received either 2% carboxymethylcellulose vehicle or 30 mg/Kg of CMC 2.24 by oral intubation daily for 2 weeks. Contralateral LPS (3 *μ*L, 30 *μ*g) or PBS (3 *μ*L) vehicle injections were performed 3 times/week for 14 days at the palatal aspect of first molars (see Figure 1). The bar graph presents the results of the *μ*-CT analysis as the change in the bone volume fraction (BVF) in the standardized ROI (bidimensionally shown as a red box in the representative image of the control) in comparison to vehicle-treated/PBS-injected samples (BVF in these samples was set to 100% since these were assumed to present no inflammatory bone resorption). Bars indicate average and standard deviations.  *Significant difference in comparison to PBS-injected/vehicle-treated control. Differences between experimental conditions are indicated by the brackets and ∗ (unpaired *t*-test for independent samples with Welch's correction for unequal variances). Images in (b) show three-dimensional rendering of the mineralized tissues in representative samples.

To confirm and expand these data on alveolar bone loss in the four experimental groups (Figure 1), additional measurements using *μ*-CT were carried out. As shown in Figure 4, these data again demonstrate that LPS increased the loss of bone in the AOI and that CMC 2.24 administration reduced this bone loss to the level seen in the control rats in which the gingivae were injected with PBS instead of LPS.

**Figure 5:**
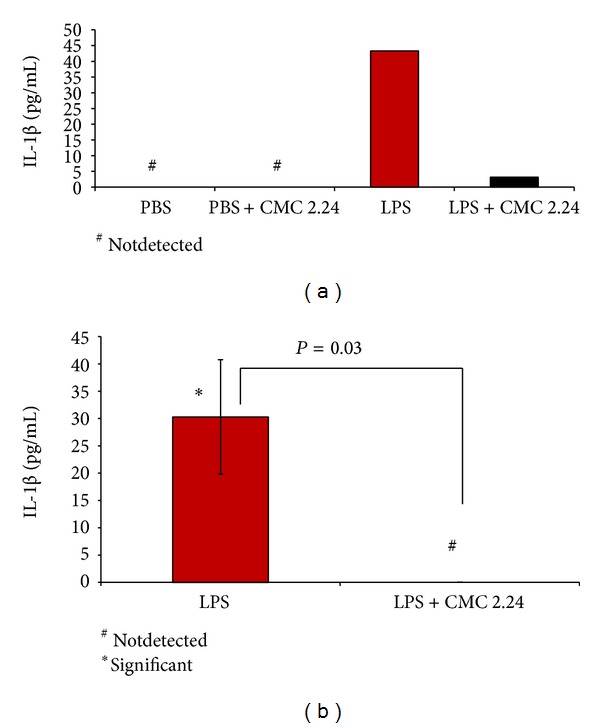
(a) The effect of CMC therapy on IL-1*β* in rat gingiva (top) and (b) serum (bottom) measured by ELISA.

Analysis of IL-1*β* in extracts of the pooled gingival tissues indicated that LPS injections markedly increased the level of this proinflammatory cytokine since it was not detectable in the extracts of the PBS-injected gingival tissue (Figure 5(a)). Moreover, CMC 2.24 administration reduced the pathologically excessive levels of IL-1*β* in the gingiva by 93% (Figure 5(a)). Similar concentrations of IL-6 were detected in the gingival tissues from the different groups of rats; however, the LPS injections did not appear to affect these levels and CMC 2.24 treatment only slightly reduced the levels of this cytokine by about 15% (data not shown). TNF-*α* was undetectable in both gingival extracts and serum (see below).

### 3.2. Systemic Measurements: Plasma and Serum

**Figure 6:**
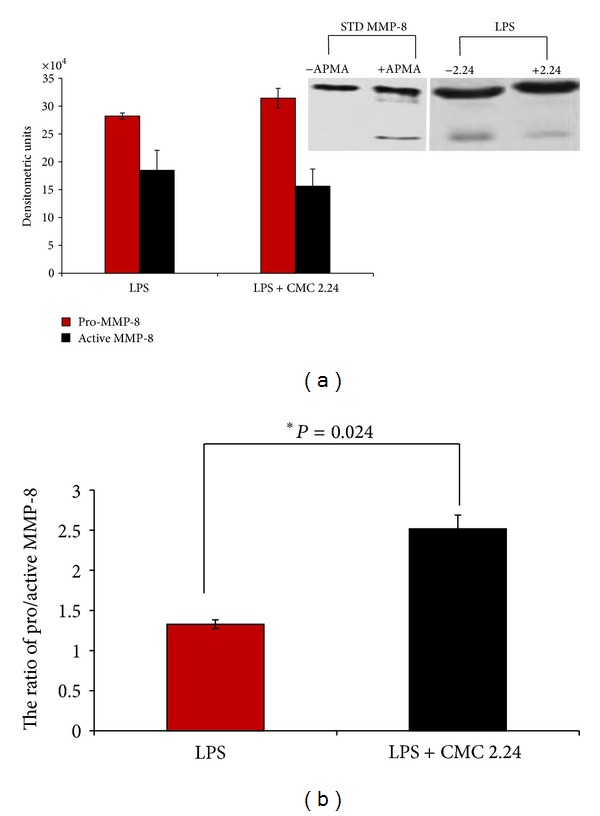
(a) Densitometric analysis of western blots of MMP-8 in plasma from untreated LPS- injected rats (LPS) and LPS-injected rats treated with CMC 2.24 (LPS + CMC 2.24). Each value represents the mean of MMP-8 ± the standard error of the mean (SEM); representative western blots of MMP-8 in plasma from untreated and CMC 2.24-treated rats are shown in the insert. (b) The ratio of pro/active MMP-8 calculated from densitometric analysis shown in (a) above.

In the experimental protocol used in the current study (a “split-mouth” design), MMP-8 (neutrophil-type collagenase, collagenase-2) and MMP-13 (collagenase-3) were both detected in the plasma samples from the different groups of rats but neither was detected in the gingiva (see Section 4). Based on western blot analysis, the plasma samples from the LPS-injected rats (half-jaw only) which were treated by oral administration of CMC 2.24 appeared to exhibit reduced levels of activated, lower molecular weight forms of MMP-8 compared to the plasma from the LPS-treated rats administered with the vehicle alone (controls) (Figure 6(a)). Based on the densitometric analysis of these western blots (Figure 6(a)), the plasma of the CMC 2.24-treated rats with LPS-induced periodontitis exhibited a ratio of pro/active MMP-8 of 2.52 ± 0.20 (SEM) which was 89.5% higher than the ratio, 1.33 ± 0.05, seen in the plasma from the vehicle-treated LPS-periodontitis rats (Figure 6(b)), and this inhibition of activation of the precursor (latent) form of MMP-8 by the CMC2.24 treatment was statistically significant (*P* = 0.024). Note that a 4-hour incubation of the standard recombinant rat MMP-8 with 1 mM APMA, a known activator of pro-MMPs in vitro [20], converted the higher molecular weight pro-MMP-8 into the smaller molecular weight activated form of this leukocyte-type collagenase (see Figure 6(a)).

The plasma levels of MMP-13 assessed by ELISA were found to be about 1.1 *μ*g/mL. Administration of CMC 2.24 to the LPS-periodontitis rats appeared to slightly reduce the levels of this collagenase in the plasma; however, this effect was not statistically significant (data not shown).

Regarding the proinflammatory cytokines in the serum (Figure 5(b)), because of the “split-mouth” design (see Figure 1), there were no serum samples from rats without gingival LPS injection. However, the levels of IL-1*β* in the serum of these LPS-exposed rats (about 30 pg/mL) were significantly (*P* = 0.03) reduced to undetectable levels by CMC 2.24 administration, a pattern similar to that seen in the gingival tissues (Figure 5(a)).

IL-6 showed higher concentrations in the serum (about 95 pg/mL) than IL-1*β* in the LPS-periodontitis rats, and, again, CMC 2.24 appeared to reduce the level of this cytokine. However, this lesser effect (about 18% reduction) was not statistically significant (data not shown).

## 4. Discussion

This paper advances a novel therapeutic strategy which uses systemically administered medications as adjunctive therapy to modulate the host response in periodontal disease (periodontal therapy has traditionally only focused on locally suppressing the pathogenic microorganisms in the oral biofilm), with applications for other chronic inflammatory diseases as well (see below). The clinical application of this strategy began with the discovery that tetracyclines (TCs), unexpectedly, can inhibit host-derived MMPs, inflammatory mediators (e.g., the cytokine IL-1*β*), and collagen degradation including bone resorption; and by mechanisms not dependent on the antibacterial properties of these drugs [4, 7–10]. Soon thereafter, doxycycline was found to be a more potent MMP-inhibitor than other tetracycline antibiotics, including minocycline and tetracycline itself, and was subsequently developed and approved as a nonantibiotic low-dose formulation for long-term administration to patients with chronic periodontitis and the dermatologic inflammatory disease, rosacea [4, 9]. Based on these earlier and the current studies, the nontetracycline chemically modified curcumin (discussed below) appears to be as, or more, potent an MMP-inhibitor compound compared to doxycycline [4, 8, 9, 13]. As one example, the IC_50_ (the concentration of the compound required to inhibit 50% of MMP activity in vitro) of doxycycline has been reported to be approximately 15 *μ*M [8, 9]. In contrast, recent studies by our group have demonstrated IC_50_ levels of CMC 2.24 at even lower *μ*M levels (2–5 *μ*M) when tested in vitro against MMPs such as MMP-8 (leukocyte-type collagenase), MMP-9 (leukocyte-type gelatinase), MMP-12 (macrophage metalloelastase), and MMP-14 (membrane-type MMP) [13]. However, a significant disadvantage of the approved subantimicrobial-dose formulations of doxycycline is that NO increase in the dose of this tetracycline can be prescribed to the patient (which might be desirable in order to, possibly, enhance the efficacy of this treatment in collagen-destructive diseases, e.g., periodontitis) because the low nonantibiotic blood levels of the drug (<1 *μ*g/mL) produced by this formulation cannot be exceeded in order to prevent an important side-effect, namely, the emergence of tetracycline-resistant or pan-antibiotic-resistant bacteria [4]. In contrast, the potential strategy of long-term administration of CMC 2.24, for inflammatory diseases, would not be undermined by this strict, low-dose, limitation because this compound is not an antibiotic like the tetracyclines.

As described earlier (see Section 1), natural curcumin has a similar active site (i.e., the diketone zinc-binding moiety) as the tetracyclines and can also modulate the host response including MMP-inhibition and suppression of inflammatory mediators [25–31], although it is ineffective against alveolar bone loss (see below). However, the chemically modified curcumin, CMC 2.24, tested in the current in vivo study, has a modified active site which is triketonic as detailed by us in previous studies by Zhang et al. [13, 14] and does effectively inhibit bone loss.

Recently, newer host-modulating medications have also been investigated as adjunctive treatment for periodontal disease and related medical disorders. These, in particular, have included (1) the resolvins such as the polyunsaturated fatty acids [32] which do not suppress the acute inflammatory response required by the host to combat infection, but which do prevent the tissue-destructive prolongation of this process, and (2) the subject of the current study, the chemically modified curcumins (CMCs). Of importance, the latter have shown improved solubility, zinc-binding, and biological effects in comparison with natural curcumin [13, 14]. Development of these CMCs is based on maintaining a similar active site for MMP-inhibition as that of the tetracyclines but with a different phenolic superstructure [11], which most recently resulted in the development of a new series of compounds with a triketonic zinc-binding moiety, which are still bicyclic rather than tetracyclic, that is, the chemically modified curcumins or CMCs. A series of these triketonic CMCs have been developed including CMC 2.5 (a methoxy carbonyl curcumin [11]) which, in turn, has been superseded by a more potent MMP-I compound, CMC 2.24, a phenylamino carbonyl curcumin; the latter has shown evidence of efficacy (and safety) in vitro, in cell and tissue culture, and in vivo models of several diseases including arthritis, diabetes, and cancer [13–15, 33].

The current study is the first to demonstrate efficacy of this compound, CMC 2.24, in an animal model of experimental periodontitis. Evidence of the onset and progression of this disease, induced by several injections of LPS into the gingiva of the rat, included dramatic increases in several forms (both pro- and activated) of connective tissue-destructive MMP-2 (72 kDa) and MMP-9 (92 kDa) gelatinases, elevated levels of the inflammatory cytokine often associated with periodontitis, IL-1*β*, and, most importantly in this model, a significant increase in alveolar bone loss, assessed morphometrically and by *μ*-CT, in the same jaws as the increase in gingival inflammatory mediators and MMPs (the impact of this local inflammatory disease and this experimental treatment on systemic levels of mediators is discussed below).

The potent efficacy of CMC 2.24 was demonstrated by (i) the statistically significant reduction of the LPS-induced, pathologically elevated alveolar bone loss down to the levels seen in the healthy controls and (ii) the essentially complete reduction of the pro- and activated, pathologically excessive levels of MMP-2, MMP-9, and IL-1*β*, in the inflamed gingival tissues back down to the un- (or barely) detectable levels seen in the control gingiva. As a result of the profound efficacy of this novel compound in this initial study, we now have a rationale to initiate studies using a modified animal model of experimental periodontal disease, which does not use “split-mouth” design, and in a periodontitis model in which the CMC 2.24 is administered therapeutically (after the disease has been established) rather than prophylactically as in the current study. In a more recent study in which alveolar bone loss was assessed at the cellular level histomorphometrically and histochemically, a similar pattern of change was seen, namely, that LPS injection increased osteoclast-mediated bone resorption and that CMC 2.24 inhibited this mechanism of alveolar bone loss (Guimaraes et al., in preparation). The potency of the biological effects of CMC 2.24 at an oral dose of 30 mg/kg is further demonstrated by the fact that, in previous experiments, we have not observed a significant decrease of inflammatory-driven bone resorption with 100 mg/kg dose of natural curcumin [16]. Interestingly, in recent experiments, we found that daily administration of 400 mg/kg of natural curcumin significantly reduced inflammatory-driven bone resorption in this model, but this dose of natural curcumin is more than 10-fold higher than the dose of CMC 2.24 administered in the current study (Guimaraes et al., in preparation).

Regarding insights into the mechanisms, plus the impact of this local disease and its treatment on the systemic condition of the host, we also observed the following: (i) the apparent reduction of IL-1*β* by CMC 2.24 treatment in the pooled gingival tissues was paralleled by a dramatic and significant reduction in this inflammatory mediator in the systemic circulation of the same animals, and (ii) for CMC 2.24 treatment, although it did not appear to alter the total levels of MMP-8 (neutrophil-type collagenase) in the blood samples of the LPS-injected rats, it did significantly reduce the ratio of the lower molecular weight, activated, collagen-destructive forms of this collagenase relative to the higher molecular weight, inactive, proforms of this MMP (note that, in the current experiment, MMP-8 could not be detected in the pooled gingival tissue). Mechanisms could include the ability of CMC 2.24 to inhibit other neutral proteinases such as plasmin, elastase, and MMP-1 which are known to cleave the amino-terminal propeptide domain of pro-MMP-8, converting it into the smaller molecular weight activated forms [9, 20]. Of relevance to the mechanisms involving CMCs ability to inhibit pro-MMP activation, recent studies (S. Simon et al., unpublished data) indicate that 2.24 can inhibit serine neutral proteinases (i.e., neutrophil elastase) which could explain the reduced conversion of pro- into smaller molecular weight activated MMPs which was observed in the current study in the systemic circulation. Still another possible mechanism involves the potential of this compound to inhibit the production of reactive oxygen metabolites (e.g., hypochlorous acid, HOCl). These are known to mediate proteinase activation by dissociating the thiol group in the propeptide domain [20]. This mechanism is significant because MMP-8 is largely derived from the degranulation of polymorphonuclear leukocytes, and, in the human periodontal pocket, MMP-8 constitutes about 80–90% of the total collagenase in this lesion; MMP-13 is the second most dominant collagenase in the periodontal pocket in humans, contributing about 10–20% of the total, and is thought to be derived from the junctional epithelium and bone cells [7, 34]. However, in the rat, MMP-13 is analogous to the constitutive collagenase, MMP-1, in humans and likely plays a role in physiologic turnover of collagen rather than the pathological degradation of collagen during periodontitis. In this regard, MMP-13 also could not be detected in the inflamed gingival tissues in the rats in the current study and, although it was detected in the plasma, was not reduced by CMC 2.24 treatment suggesting a preferential effect of the test compound on pathologically elevated rather than on constitutive levels of these MMPs. Additional mechanisms include the ability of natural curcumins to inhibit various signaling pathways and transcription factors involved in the expression of inflammatory mediators (AP-1, MAPK, NF-kB, and STAT3) resulting in a decrease in the expression of the inactive proforms of the MMPs and of inflammatory cytokines and, ultimately, a marked change in the microenvironment [25, 35, 36].

## 5. Conclusions

The results of this initial study indicate that the oral administration of a novel, triketonic phenylamino carbonyl curcumin (CMC 2.24), to rats with endotoxin- (LPS-) induced periodontitis, is a significant and potent inhibitor of both pathologic alveolar bone loss and its inflammatory and collagen-destructive mediators. Moreover, this chemically modified curcumin appears to have additional benefits by reducing the impact of this local inflammatory disease on systemic biomarkers of the host without (apparently) negatively affecting the mediators of constitutive connective tissue turnover. Studies are now underway to expand these observations in additional rat models of experimental inflammatory periodontal disease with a particular focus on CMC 2.24 effects (i) on the cellular mechanisms of alveolar bone loss; (ii) in a model in which the test medication is administered therapeutically (i.e., after the disease has been established) rather than prophylactically; and (iii) on the pharmacokinetics (such as peak blood levels; serum half-life) of this novel compound.

## References

[1] Hu J, Van den Steen PE, Sang QA, Opdenakker G (2007). Matrix metalloproteinase inhibitors as therapy for inflammatory and vascular diseases. *Nature Reviews Drug Discovery*.

[2] Sorsa T, Tjäderhane L, Konttinen YT (2006). Matrix metalloproteinases: contribution to pathogenesis, diagnosis and treatment of periodontal inflammation. *Annals of Medicine*.

[3] Overall CM, López-Otín C (2002). Strategies for MMP inhibition in cancer: innovations for the post-trial era. *Nature Reviews Cancer*.

[4] Gu Y, Walker C, Ryan ME, Payne JB, Golub LM (2012). Non-antibacterial tetracycline formulations: clinical applications in dentistry and medicine. *Journal of Oral Microbiology*.

[5] Frankwich K, Tibble C, Torres-Gonzalez M (2012). Proof of concept: matrix metalloproteinase inhibitor decreases inflammation and improves muscle insulin sensitivity in people with type 2 diabetes. *Journal of Inflammation*.

[6] Engebretson SP, Hey-Hadavi J (2011). Sub-antimicrobial doxycycline for periodontitis reduces hemoglobin A1c in subjects with type 2 diabetes: a pilot study. *Pharmacological Research*.

[7] Golub LM, Lee HM, Greenwald RA (1997). A matrix metalloproteinase inhibitor reduces bone-type collagen degradation fragments and specific collagenases in gingival crevicular fluid during adult periodontitis. *Inflammation Research*.

[8] Golub LM, Ramamurthy NS, McNamara TF, Greenwald RA, Rifkin BR (1991). Tetracyclines inhibit connective tissue breakdown: new therapeutic implications for an old family of drugs. *Critical Reviews in Oral Biology and Medicine*.

[9] Golub LM, Lee HM, Ryan ME, Giannobile WV, Payne J, Sorsa T (1998). Tetracyclines inhibit connective tissue breakdown by multiple non-antimicrobial mechanisms. *Advances in dental research*.

[10] Payne JB, Golub LM (2011). Using tetracyclines to treat osteoporotic/osteopenic bone loss: from the basic science laboratory to the clinic. *Pharmacological Research*.

[11] Gu Y, Lee HM, Napolitano N (2013). 4-methoxycarbonyl curcumin: a unique inhibitor of both inflammatory mediators and periodontal inflammation. *Mediators of Inflammation*.

[12] Amin AR, Attur M, Abramson SB (1999). Nitric oxide synthase and cyclooxygenases: distribution, regulation, and intervention in arthritis. *Current Opinion in Rheumatology*.

[13] Zhang Y, Gu Y, Lee HM (2012). Design, synthesis and biological activity of new polyenolic inhibitors of matrix metalloproteinases: a focus on chemically-modified curcumins. *Current Medicinal Chemistry*.

[14] Zhang Y, Golub LM, Johnson F, Wishnia A (2012). PKa, Zinc- and serum albumin-binding of curcumin and two novel biologically-active chemically-modified curcumins. *Current Medicinal Chemistry*.

[15] Botchkina GI, Zuniga ES, Rowehl RH (2013). Prostate cancer stem cell-targeted efficacy of a new-generation taxoid , SBT-1214 and novel polyenolic zinc-binding curcuminoid, CMC2.24. *PLoS ONE*.

[16] Guimarães MR, Coimbra LS, De Aquino SG, Spolidorio LC, Kirkwood KL, Rossa C (2011). Potent anti-inflammatory effects of systemically administered curcumin modulate periodontal disease *in vivo*: curcumin inhibits periodontal disease *in vivo*. *Journal of Periodontal Research*.

[17] Payne JB, Golub LM, Stoner JA (2011). The effect of subantimicrobial-dosedoxycycline periodontal therapy on serum biomarkers of systemic inflammation: A randomized, double-masked, placebo-controlled clinical trial. *Journal of the American Dental Association*.

[18] Garcia de Aquino S, Manzolli Leite FR, Stach-Machado DR, Francisco da Silva JA, Spolidorio LC, Rossa C (2009). Signaling pathways associated with the expression of inflammatory mediators activated during the course of two models of experimental periodontitis. *Life Sciences*.

[19] Ramamurthy NS, Golub LM (1983). Diabetes increases collagenase activity in extracts of rat gingiva and skin. *Journal of Periodontal Research*.

[20] Golub LM, Evans RT, McNamara TF, Lee HM, Ramamurthy NS (1994). A non-antimicrobial tetracycline inhibits gingival matrix metalloproteinases and bone loss in Porphyromonas gingivalis-induced periodontitis in rats. *Annals of the New York Academy of Sciences*.

[21] Lee H, Ciancio SG, Tüter G, Ryan ME, Komaroff E, Golub LM (2004). Subantimicrobial dose doxycycline efficacy as a matrix metalloproteinase inhibitor in chronic periodontitis patient is enhanced when combined with a non-steriodal anti-inflammatory drug. *Journal of Periodontology*.

[22] Brown DL, Desai KK, Vakili BA, Nouneh C, Lee H, Golub LM (2004). Clinical and biochemical results of the metalloproteinase inhibition with subantimicrobial doses of doxycycline to prevent acute coronary syndromes (MIDAS) pilot trial. *Arteriosclerosis, Thrombosis, and Vascular Biology*.

[23] de Souza DM, de Prado FA, de Prado MA, da Rocha RF, de Carvalho YR (2010). Evaluation of two morphometric methods of bone loss percentages caused by periodontitis in rats in different locations. *Journal of Applied Oral Science*.

[24] Golub LM, Sorsa T, Lee HM (1995). Doxycycline inhibits neutrophil (PMN)-type matrix metalloproteinases in human adult periodontitis gingiva. *Journal of Clinical Periodontology*.

[25] Bharti AC, Takada Y, Aggarwal BB (2004). Curcumin ( diferuloylmethane ) inhibits receptor activator of NF- *κ*B ligand-induced NF- *κ*B activation in osteoclast precursors and suppresses osteoclastogenesis. *The Journal of Immunology*.

[26] Grynkiewicz G, Ślifirski P (2012). Curcumin and curcuminoids in quest for medicinal status. *Acta Biochimica Polonica*.

[27] Woo M, Jung S, Kim S (2005). Curcumin suppresses phorbol ester-induced matrix metalloproteinase-9 expression by inhibiting the PKC to MAPK signaling pathways in human astroglioma cells. *Biochemical and Biophysical Research Communications*.

[28] Banerji A, Chakrabarti J, Mitra A, Chatterjee A (2004). Effect of curcumin on gelatinase a (MMP-2) activity in B16F10 melanoma cells. *Cancer Letters*.

[29] Shakibaei M, John T, Schulze-Tanzil G, Lehmann I, Mobasheri A (2007). Suppression of NF-*κ*B activation by curcumin leads to inhibition of expression of cyclo-oxygenase-2 and matrix metalloproteinase-9 in human articular chondrocytes: implications for the treatment of osteoarthritis. *Biochemical Pharmacology*.

[30] Kaur G, Tirkey N, Bharrhan S, Chanana V, Rishi P, Chopra K (2006). Inhibition of oxidative stress and cytokine activity by curcumin in amelioration of endotoxin-induced experimental hepatoxicity in rodents. *Clinical and Experimental Immunology*.

[31] Begum AN, Jones MR, Lim GP (2008). Curcumin structure-function, bioavailability, and efficacy in models of neuroinflammation and Alzheimer's disease. *Journal of Pharmacology and Experimental Therapeutics*.

[32] Serhan CN, Chiang N, Van Dyke TE (2008). Resolving inflammation: dual anti-inflammatory and pro-resolution lipid mediators. *Nature Reviews Immunology*.

[33] Katzap E, Goldstein MJ, Shah NV (2011). The chondroprotective properties of curcumin (*Curcuma longa*) and curcumin derived polyenolic zinc binding inhibitors against IL-1*β* and OsM -induced chrondrolysis. *Transactions of the Orthopedic Research Society*.

[34] Golub LM, Lee HM, Stoner JA (2010). Doxycycline effects on serum bone biomarkers in post-menopausal women. *Journal of Dental Research*.

[35] Guimarães MR, de Aquino SG, Coimbra LS, Spolidorio LC, Kirkwood KL, Rossa C (2012). Curcumin modulates the immune response associated with LPS-induced periodontal disease in rats. *Innate Immunity*.

[36] de Souza JAC, Rossa Junior C, Garlet GP, Nogueira AVB, Cirelli JA (2012). Modulation of host cell signaling pathways as a therapeutic approach in periodontal disease. *Journal of Applied Oral Science*.

